# Long-Term Outcomes of the Flixene Vascular Graft Used for Haemodialysis

**DOI:** 10.7759/cureus.13459

**Published:** 2021-02-20

**Authors:** Bulang He, Duxxa Tailor, Zi Qin Ng, Shaun Samuelson, Sanjay Nadkarni, Matt Van Myk, John Ferguson, Jonathan Tibballs, Doris Chan

**Affiliations:** 1 Western Australia (WA) Liver and Kidney Transplant Service, Sir Charles Gairdner Hospital, University of Western Australia, Perth, AUS; 2 Department Renal Surgery and Transplant, Austin Hospital, Victoria, AUS; 3 Department Renal Surgery and Transplant, Alfred Hospital, Monash University, Prahran, AUS; 4 Western Australia (WA) Liver and Kidney Transplant Service, Sir Charles Gairdner Hospital, Perth, AUS; 5 Department of Radiology, Sir Charles Gairdner Hospital, Perth, AUS; 6 Department of Nephrology, Sir Charles Gairdner Hospital, Perth, AUS

**Keywords:** flixene graft, arteriovenous fistula, arteriovenous graft fistula, haemodialysis access, primary patency rate, secondary patency rate, central venous catheter (cvc)

## Abstract

Introduction

The Flixene™ vascular graft (Maquet-Atrium Medical, Hudson, NH) has emerged as a new generation synthetic graft, which allows for early needling for haemodialysis. Most studies have shown satisfactory early results. The aim of this study is to report on long-term outcomes of the Flixene graft over eight years in a cohort of patients.

Methods

From February 2011 to September 2019, 61 patients had 64 arteriovenous graft fistulas (AVGs) by using the Flixene grafts. The median patient age was 67 years; the male to female ratio was 30:31. Diabetes was the reason for the majority of kidney failures (57%). The configuration of the grafts used was mostly upper arm straight AVG. The primary patency rate, secondary patency rate, and surgical complications were assessed.

Results

In a median follow-up of three years (interquartile range (IQR): 2 - 6), 36 of the AVGs required a fistulogram. Venous side stenosis was the most common cause of high venous pressure or AVG occlusion in 97%. The one-year primary patency rate was 30%. The secondary patency rate was 94.8%, 83.7%, and 77.7% at one, three, and five years, respectively. The longest functional AVG was observed for up to seven years.

Conclusions

This study has shown satisfactory long-term results of the Flixene graft used for hemodialysis. The Flixene graft could be needled within 72 hours without increased complications, which allows the creation of an AVG under an emergency setting to avoid the placement of a central venous catheter (CVC). This strategy should be advocated in future clinical practice.

## Introduction

A native arteriovenous fistula (AVF) is the permanent access of choice in patients who require haemodialysis (HD) as it has been associated with a lower risk of infection, less thrombosis, and a better patency rate [1-4]. Unfortunately, in the past decade, a decline has been seen in the primary and secondary patency rates of AVF, with approximately one-third of newly formed AVFs failing to mature [1- 2]. Arteriovenous graft (AVG) fistula is an alternative in patients without suitable veins for an AVF. Although AVGs are prone to infection and thrombosis in comparison with AVFs [3-4], they achieve a better outcome than a central venous catheter (CVC) as the latter is associated with the highest risk of infection and its associated morbidities [5-8].

Over the last decade, new generation vascular grafts have been developed that could be needled earlier within 72 hours after placement, such as the Flixene™ graft (Maquet-Atrium Medical, Hudson, NH) [9], potentially negating the need for CVC placement and allowing for earlier removal of the CVC. The early- and mid-term outcomes have been shown to be satisfactory [10-12]. However, studies of long-term outcomes are lacking. Therefore, the aim of this study is to report on the long-term outcomes of the Flixene graft over an eight-year follow-up in our cohort of patients.

## Materials and methods

The study was conducted by retrospectively analyzing the prospectively collected data in a single-center involving a cohort of patients who required a synthetic vascular graft for the creation of haemodialysis access between February 2011 and September 2019 at Sir Charles Gairdner Hospital, Perth, Australia. The study was approved as a Quality Improvement Project (#QI 16136) by the local Institutional Human Research Ethics Committee of Sir Charles Gairdner Hospital. 

The indication for using a synthetic vascular graft was that the patients were lacking suitable veins for the creation of a native AVF. The assessment included clinical examination, Doppler ultrasound of the vein mapping, and venography.

Surgical technique

For an AVG at the Upper Arm (Straight Configuration)

A small incision was made at the elbow for brachial artery dissection. A second small incision was made in the axillary area for axillary vein access. A Flixene graft was placed subcutaneously at the upper arm by using a slightly curved tunneler (Maquet-Atrium Medical, Hudson, NH). The venous end of the graft was anastomosed first in an end-to-side fashion to the axillary vein, then the arterial end was anastomosed in the same fashion to the side of the brachial artery by using 6/0 Prolene®. The graft was flushed with heparinised normal saline (1000 IU of heparin in 100 ml of normal saline) prior to the completion of each anastomosis.

For the Creation of AVG at the Thigh (Loop Configuration)

An incision was made in the groin area parallel to the inguinal ligament and two finger's breadth inferior. The femoral artery and femoral vein were dissected and looped. Firstly, one end of the Flixene graft was anastomosed to the side of the femoral vein, then the graft was tunneled in a loop fashion by a curved tunneler with a small incision made at the apex of the loop at mid-thigh. The other end of the graft was brought to the same groin incision by the curved tunneller meeting the femoral artery and was anastomosed to the side of the femoral artery using 6-0 Prolene. The graft was flushed with heparinised normal saline (1,000 IU of heparin in 100 ml of normal saline) prior to the completion of each anastomosis.

The blood flow in the AVG was assessed by a handheld Doppler device at the end of the procedure. Overall, two Flixene intraluminal flow guard (IFG) grafts (4-7 mm x 40 cm) and 63 Flixene grafts (6 mm x 40 cm) were used in this cohort. One Flixene IFG graft was replaced by a Flixene graft during the procedure immediately following the completion of anastomoses for concerns of poor flow.

Surveillance program

All patients were routinely educated for self-monitoring of AVG function daily. The functional status of the AVG was assessed when on HD. The vascular access nurse specialist collaborated with the dialysis nurses to monitor the AVG through dialysis outcomes and clinical assessment. Monitoring through dialysis outcomes included measurement of flows and pressures, particular attention to the trends in venous pressure, clearances as measured by online KT/V, flow monitoring, and recirculation studies. Clinical assessment included prolonged needle site bleeding time, arm swelling, and changes in thrill and bruit. Any concerns that arose from the dialysis were referred to the vascular access nurse specialist for further assessment and the multidisciplinary team review of the prior imaging. This was followed by a referral to interventional radiology (IR) for fistulogram ± angioplasty as a semi-urgent procedure. All AVG occlusions were primarily referred to IR for endovascular thrombectomy and fistulogram ± angioplasty as an emergency procedure. The surgical team was kept informed of the outcomes. Surgical open thrombectomy was performed only in the cases that failed the IR procedure. Then, the subsequent fistulogram ± angioplasty were routinely performed following open surgical thrombectomy to treat any underlying venous side stenosis.

The surveillance program also included the patients who had confirmed stenosis on fistulogram, for which a three-monthly fistulogram ± angioplasty were performed until a proven resolution on updated fistulogram.

The primary outcomes were to assess the one-year primary patency rate and the secondary patency rate during follow-up. The primary patency rate is defined as from the time of AVG creation to any first intervention to maintain or restore the function of the AVG or reaching a censored event (death, transplantation, conversion to peritoneal dialysis, and end of the study period). The secondary patency rate is defined as from the time of the AVG creation until AVG abandonment or achievement of a censored event (death, transplantation, conversion to peritoneal dialysis, and end of the study period) [13-15]. 

The secondary outcomes were to evaluate surgical complications and long-term graft function. The AVG functional rate is defined as the same as the secondary patency rate in this study. This is due to all the AVGs in this cohort that were used for hemodialysis until abandonment as a result of the unsalvageable condition of occlusion or achieving a censored event (death, kidney transplant, conversion to peritoneal dialysis, and the end of the study). The AVG functional rate and secondary patency rate is presented using Kaplan-Meier statistical method.

Data collection

Data were extracted from the prospectively collected database included patient characteristics of gender and age, primary kidney disease, dialysis status, the configuration of the AVG, surgical complications, episode of AVG occlusion, the requirement for fistulogram or angioplasty, and the frequency for fistulogram and angioplasty.

Statistical analysis

Data were expressed as number (proportion), mean ± standard deviation for normally distributed continuous variables, and as the median and interquartile range (IQR) for non-normally distributed data. The Kaplan-Meier analysis was conducted for measurement of AVG secondary patency rate and functional rate. R Core Team (2019) (R Foundation for Statistical Computing, Vienna, Austria) was used for statistical analysis.

## Results

Sixty-one patients underwent 64 AVG creations by using the Flixene vascular grafts (Table 1). The age of the patients ranged from 22 to 88 years (median: 67 years; IQR: 55 - 75) and the male-to-female ratio was 30:31. The majority of renal failure was due to diabetes (57%), followed by 19.4% with glomerulonephritis, 11.5% due to urological disease, 4.9% due to renal vascular disease, 3.3% due to multiple myeloma, and 3.3% due to an unknown cause.

**Table 1:** Demographics and Characteristics of the Patients in this Cohort AVG: arteriovenous graft fistula; CVC: central venous catheter; IQR: interquartile range; PD: peritoneal dialysis

Demographics and Characteristics of the Cohort Patients	
Male (n, %)	30 (49%)
Female (n, %)	31 (51%)
Age (years, median IQR)	67 (55 - 75)
Primary kidney disease (n, %):	
Diabetes	35 (57%)
Glomerulonephritis	12 (19.4%)
Urological disease	7 (11.5%)
Renal vascular disease	3 (4.9%)
Multiple myeloma	2 (3.3%)
Unknown cause	2 (3.3%)
On hemodialysis by CVC	41(67%)
Previous native AVF failure	9 (14.8%)
Conversion from PD	5 (8.2%)
For second AVG	5 (8.2%)
For third AVG	1 (1.7%)
Pre-dialysis	10 (16.4%)
Straight configuration (n, %)	
Left upper arm	51 (79.7%)
Right upper arm	12 (18.8%)
Loop configuration (n, %)	
Left thigh	1 (1.6%)
Infection:	3 cases (4.8%)
	-1 (3 weeks)
	-1 (9 months)
	-1 (23 months)
Steal syndrome	1 (1.6%)
AVG pseudo-aneurysm	2 (3.1%)
Fistulograms:	36 (56%)
	-23 AVGs (due to increase in venous pressure)
	-13 AVGs (due to AVG occlusion)
Follow-up (years, median, IQR)	3 (2 - 6)

Forty-one patients (67%) were using CVC for HD prior to AVG, 14.8% of patients with a history of native AVF failure, 8.2% of patients on peritoneal dialysis for the conversation to HD, 8.2% of patients for second AVG (4.9% of them had their first AVG prior to the study period), and 1.7% patient for the third AVG. Ten patients (16.4%) were pre-dialysis.

**Figure 1:**
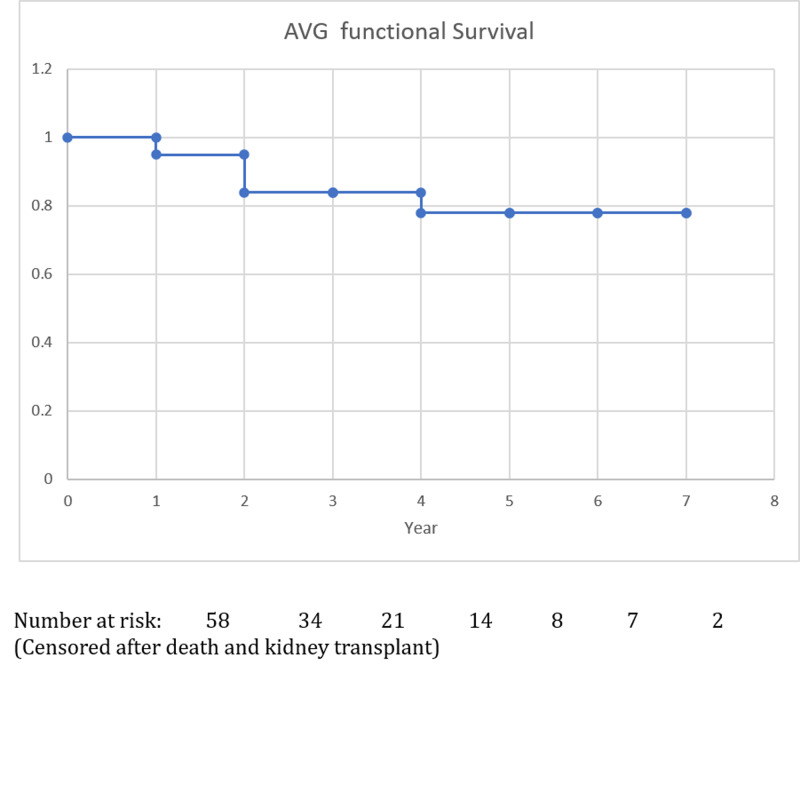
Arteriovenous graft fistula (AVG) functional rate

All AVGs were created successfully, except one Flixene IFG graft, which was replaced by a 6 mm x 40 cm Flixene graft due to poor flow detected by the Doppler device during the procedure. Sixty-three AVGs were in straight configuration at the upper arm with 51 on the left side and 12 on the right side, whereas one AVG was in a loop configuration at the left thigh. All cases were followed up from two months to eight years with a median of three years (IQR: 2 - 6 years). During the follow-up, 19 patients died of other medical comorbidities and six patients received kidney transplants. The primary patency rate was 30% at one year. The secondary patency rates (functional rates) were 94.8%, 83.7%, and 77.7% at one, three, and five years, respectively (Table 2, Figure 1). The longest functional AVG was observed for up to seven years.

**Table 2:** Arteriovenous Graft Fistula (AVG) Functional Rate

Year	1	2	3	4	5	6	7
Functional rate (%)	94.8	83.7	83.7	77.7	77.7	77.7	77.7

With regards to secondary outcomes, early thrombosis and occlusion occurred in two cases within the first 24 hours. One required surgical intervention by disconnecting the arterial end anastomosis, thrombectomy, and re-anastomosis, whereas the other case underwent an IR procedure in which a narrowed area proximal to the venous anastomotic site was seen. Balloon dilatation was subsequently performed, and a stent was placed. The AVG function of both was satisfactory thereafter. One AVG occluded two weeks post-surgery and the patient refused further intervention. Infection occurred in three cases at three weeks, nine months, and 23 months, respectively, for which the grafts were removed (4.8%). One graft was removed due to severe steal syndrome four weeks after the AVG creation (1.6%). Two AVGs were needled earlier for HD within 72 hours after creation due to a late referral to the surgical team for dialysis access and avoided the use of CVC. During the follow-up period, 36 AVGs required a fistulogram with 23 cases due to elevated venous pressure over 200 mmHg on HD, whereas 13 due to an AVG occlusion. On the fistulogram, 35 cases (97%) had venous side stenosis, while one case (3%) showed arterial side stenosis. The time for the first fistulogram was from one day to 25 months (median: 6 months, IQR: 4 - 12 months) post-AVG creation. The frequency required for fistulogram ranged from one to 16 times (median: 4 times, IQR: 3 - 9). For the treatment of AVG occlusion, seven cases were managed by IR, whereas six required surgical thrombectomies followed by fistulogram and angioplasty. The first-line treatment for venous stenosis was balloon dilatation. Stent placement was considered if the frequent recurrence (< three months) of stenosis occurred. One case required surgical revision for venous stenosis. Two AVGs required segmental replacement due to a pseudo-aneurysm two years post-AVG creation.

## Discussion

Vascular grafts have played an important role in the establishment of HD access as one-third of newly created AVFs failed to mature [1-2]. Patients using an AVG for HD have been shown to have better patient survival compared with those dependent on CVC, as the latter is associated with the greater risk of bacteremia and its associated morbidities [5-8, 16-18]. Therefore, the AVG should be considered in patients who are deemed to be at high risk of AVF failure to avoid placement of CVC or prolonged use of CVC [19-20]. The “catheter last” should be regarded as a wise strategy and an AVG should be considered as the right fistula for the right patient at the right time on the right side (4R) [21-23].

Over the last decade, new generation vascular grafts have emerged for early cannulation within 72 hours after placement, including AVflo™ (Nicast Ltd., Global Park Lod, Israel), VECTRA® (Bard Peripheral Vascular, Inc., Tempe, AZ, USA), GORE® ACUSEAL (WL Gore & Associates, Inc., Flagstaff, AZ, USA), and Flixene (Maquet-Atrium Medical, Hudson, NH, USA). A recent literature review has shown that early cannulation of these AVGs was satisfactory without an increase in complications and jeopardization of the long-term patency rate [9, 12, 24].

The Flixene vascular graft is a composite trilaminate structure graft that offers exceptional strength and durability allowing early needling. Early studies have reported satisfactory results in comparison with conventional grafts, which were usually cannulated after two to three weeks post-implantation [10-12, 24-25].

In this cohort, two Flixene grafts were needled earlier within 72 hours without increasing any complications, although the majority of the AVGs commenced use three to four weeks after creation. This was due to a few reasons. Firstly, there was no demand for early use as the most of patients (67%) in this cohort had a CVC line prior to HD. Secondly, dialysis nurses were reluctant to insert the needle into the newly placed graft in the early post-surgery period due to some extent of arm oedema and wound healing. However, the new generation vascular grafts provide an opportunity for early needling in the setting of the emergency for HD. The dialysis nurses should be educated with the information of new generation vascular grafts and encouraged to perform early cannulation to the Flixene graft. This approach could avoid the need for CVC placement or reduce the duration of CVC placement if already in situ. This concept should be advocated in future clinical practice as a strategy to prevent CVC placement and its associated morbidities [9, 11-12, 24].

In this study, it was also observed that the use of the Flixene graft for AVGs achieved satisfactory long-term results. The one, three, and five-year functional rates were 94.8%, 83.7%, and 77.7%, respectively, which are comparable to the native AVF patency rate [1]. From our experience, it was recognized that the establishment of an active surveillance program is fundamental with a dedicated vascular access nurse specialist leading the program. If an AVG developed stenosis and was addressed with an angioplasty, then this AVG would stay on the surveillance list for a three-monthly fistulogram until there was a proven resolution of the stenosis. A monthly review of this surveillance list was conducted at a multidisciplinary team meeting. This management may have prevented AVG occlusion and reduced hospitalization and the requirement for major procedures for salvage of the AVG [26].

In this study, we had the opportunity to observe the longer durability of the Flixene graft for up to seven years. On the other hand, a couple of Flixene grafts required segmental repair as a result of aneurysmal formation about two years after the graft placement. This could be attributed to repeatedly needling at the same site. Therefore, education for dialysis nurses regarding the needling technique is paramount for preserving the graft. It should be emphasized that the rope ladder technique is preferred for the insertion of the needle. This maneuver could minimize the risk of graft erosion and pseudoaneurysm formation. Understandably, AVG is associated with an increased risk of infection, given the presence of a foreign body. In this cohort, the overall infection rate of the Flixene graft was 4.8%, slightly lower than what was reported in the literature [27].

It is well-known that the common issue of AVGs is the development of venous side stenosis because of venous intimal hyperplasia which requires regular interventional procedures, as observed in this cohort [28]. It has been postulated that the turbulent flow, wall shear stress, and endothelial injury play a key role in the development of venous intimal hyperplasia and subsequent venous stenosis [26, 29-30]. While several studies have suggested modifying the venous side geometry to mitigate the intimal hyperplasia using a vein cuff, cuffed expanded polytetrafluoroethylene (ePTFE), IFG graft, and interrupted clipping device, none of these have achieved satisfactory results [29]. Future researchers should focus on the techniques that would modify the venous site anastomosis and minimize the shear force and subsequent endothelial hyperplasia.

## Conclusions

In conclusion, our study has shown that the long-term functional rates and durability of the Flixene graft are satisfactory when used for HD access The Flixene graft is also safe to be needled within 72 hours after implantation, which allows the creation of an AVG under an emergency for HD to avoid the use of CVC. This strategy should be explored in future clinical practice when considering hemodialysis access. We would support the “4R” principle when deciding on HD access to ensure the best practice of patient-centered care.
